# Contribution of Intramyocellular Lipids to Decreased Computed Tomography Muscle Density With Age

**DOI:** 10.3389/fphys.2021.632642

**Published:** 2021-06-30

**Authors:** Nicholas A. Brennan, Kenneth W. Fishbein, David A. Reiter, Luigi Ferrucci, Richard G. Spencer

**Affiliations:** ^1^Laboratory of Clinical Investigation, NIA, NIH, Baltimore, MD, United States; ^2^Department of Radiology and Imaging Sciences, Emory University School of Medicine, Atlanta, GA, United States; ^3^Longitudinal Studies Section, NIA, NIH, Baltimore, MD, United States

**Keywords:** muscle, aging, proton magnetic resonance spectroscopy, intramyocellular lipids, computerized tomography

## Abstract

Skeletal muscle density, as determined by computed tomography (CT), has been shown to decline with age, resulting in increased frailty and morbidity. However, the mechanism underlying this decrease in muscle density remains elusive. We sought to investigate the role of intramyocellular lipid (IMCL) accumulation in the age-related decline in muscle density. Muscle density was measured using computerized tomography (CT), and IMCL content was quantified using *in vivo* proton magnetic resonance spectroscopy (^1^H-MRS). The study population consisted of 314 healthy participants (142 men, 32–98 years) of the Baltimore Longitudinal Study of Aging (BLSA). In addition to IMCL quantification, obesity-related covariates were measured, including body mass index (BMI), waist circumference, and circulating triglyceride concentration. Higher IMCL concentrations were significantly correlated with lower muscle density in older individuals, independent of age, sex, race, and the obesity-associated covariates (*p* < 0.01). Lower muscle density was also significantly associated with greater age-adjusted IMCL, a variable we constructed using LOESS regression (*p* < 0.05). Our results suggest that the accumulation of IMCL may be associated with a decrease in muscle density. This may serve to define a potential therapeutic target for treatment of age-associated decreased muscle function.

## Introduction

Skeletal muscle radiological density, as assessed with computerized tomography (CT), has been shown to decrease with age (Lauretani et al., 2006). Independent of age and other potential confounders, lower muscle density has been associated with frailty (Cesari et al., 2006), mobility limitations (Visser et al., 2005; McDermott et al., 2009), increased risk of hip fracture (Lang et al., 2010), poor lower extremity performance (Visser et al., 2002; Hicks et al., 2005; Cawthon et al., 2009), and lower muscle quality, defined as the specific force generated per unit of muscle volume (Goodpaster et al., 2001a; Conroy et al., 2012). In addition, lower muscle density has been associated with metabolic outcomes such as loss of oxidative enzyme capacity (Schrauwen-Hinderling et al., 2007), insulin resistance, the metabolic syndrome and higher intermuscular fat deposition (Goodpaster et al., 2000a, 2001a; Visser et al., 2005; Addison et al., 2014). Based on these data, it has been suggested that low muscle density is a biomarker of impaired metabolism and poor health status.

In spite of the consistent observation that muscle density declines with age, and the strong relationship between poor muscle density and adverse health outcomes (Pahor and Kritchevsky, 1998), the underlying mechanisms that cause reduction in muscle density remain poorly understood. Central hypotheses point to accumulation of small adipose cell aggregates that cannot be resolved by CT, but which affect overall tissue density. At the microscopic level, accumulation of lipids occurs mainly in two distinct compartments. First, intramyocellular lipid (IMCL) represents fat stored in the form of cytoplasmic droplets within myocytes; this can occur in response to metabolic derangements, including mitochondrial dysfunction. IMCL tends to be uniformly distributed throughout the muscle, and is particularly high in diabetic and obese individuals (Schrauwen-Hinderling et al., 2006; Weis et al., 2007; Aguer et al., 2010; Brumbaugh et al., 2012; Bredella et al., 2013; Noble et al., 2014). In contrast, extramyocellular lipids (EMCL) are layers of fat deposited outside myocytes. Unlike IMCL, EMCL is largely metabolically inert, and its quantification is highly dependent on voxel orientation and placement (Boesch et al., 1997). Therefore, our study focuses on IMCL deposition in aging muscle. While neither IMCL nor EMCL can be directly visualized with CT, macroscopic fat can be readily delineated. Additionally, large amounts of macroscopic fat can lead to a high EMCL signal, obscuring the ability to accurately discriminate between these two depots of fat using magnetic resonance spectroscopy.

Infiltration of muscle with fat is well-known to indicate decreased muscle quality. This can be observed with CT, with fat-infiltrated muscle demonstrating decreased attenuation as compared to non-fatty muscle (Prasetyo et al., 2020). However, this attenuation is not specific to the most metabolically significant fat component, IMCL. In contrast, MR spectroscopy provides a means to directly assess IMCL. Unfortunately, MRS is a highly specialized technique which is not widely available outside of medical research centers. Thus, we evaluate the degree to which IMCL contributes to the decreased CT attenuation exhibited by fat-infiltrated muscle. This provides insight into the degree to which CT evaluation of muscle can indicate decreased muscle quality as defined by IMCL infiltration.

The metabolic role of IMCL is recognized as providing a readily accessible energy reserve during high-demand exercise and, consistently, IMCL has been shown to increase with high-intensity training (Goodpaster et al., 2001b). Conversely, accumulation of intramyocellular fat droplets in other settings is attributed to the relative inability of dysfunctional mitochondria to fully process lipid substrates through beta oxidation. In accordance with this view, increased deposition of IMCL has been associated with oxidative stress and glucose intolerance. In addition, increased IMCL has been shown to correlate with insulin resistance in the obese (Pan et al., 1997; Goodpaster et al., 2000b) and older populations (Petersen et al., 2003; Cree et al., 2004; St-Onge, 2005; Nakagawa et al., 2007). Individuals with metabolic syndrome, characterized in part by insulin resistance, tend to have high IMCL (Perseghin, 2005; Ingram et al., 2011; Yokota et al., 2013). Finally, compared to normal weight individuals, obese patients with high IMCL exhibit decreased muscle force, diminished myofibril contraction rate, and decreased power production (Choi et al., 2016).

Studies of IMCL and EMCL have been greatly enhanced by application of magnetic resonance spectroscopy (MRS) and imaging (MRI) (van der Meer et al., 2012; Machann et al., 2013). Proton MRS (^1^H-MRS) is currently the leading technique for quantifying IMCL in skeletal muscle. These fat compartments can be distinguished from each other based on the small difference in resonance frequency they exhibit within the magnetic field of an MR system (Schick et al., 1993; Boesch, 2007), leading to their visualization as resolved, or partly-resolved, resonances in high-quality ^1^H-MRS spectra.

Specifically defining muscle density as the mean CT attenuation coefficient of mid-thigh muscle tissue, we hypothesized that lower muscle density would be associated with accumulation of IMCL, rendering density a potential indicator of metabolic deficits. 1H-MRS has been used extensively to quantify IMCL and EMCL in human subjects (Schick et al., 1993; Boesch et al., 1997; Jacob et al., 1999; Krssak et al., 1999; Hwang et al., 2001; Howald et al., 2002; Larson-Meyer et al., 2002), while CT muscle density has been incorporated into many studies of aging (Goodpaster et al., 2001a; Anderson et al., 2013; Aubrey et al., 2014; Kalyani et al., 2014). For example, Goodpaster et al. (2001a) found that higher muscle density is associated with higher specific force production and lower BMI, while (Aubrey et al., 2014) found that lower muscle attenuation is correlated with cancer progression and poor health outcomes.

Accordingly, in this study we sought to investigate the association between muscle density, as determined by CT (Goodpaster et al., 2001a; Visser et al., 2005), and IMCL, as determined by ^1^H-MRS using data collected from subjects enrolled in the Baltimore Longitudinal Study on Aging (Ferrucci, 2008), a well-characterized cohort of normatively aging adults. Specifically, we tested the hypothesis that the decrease in muscle density associated with aging is accounted for, at least in part, by the accumulation of IMCL. In addition, due to the documented association between IMCL and obesity, we investigated the individual correlations between IMCL and several markers of obesity, including BMI itself (Amin et al., 2015), serum cholesterol concentration (Miettinen, 1971; Kurata et al., 1990), waist circumference (Brumbaugh et al., 2012; Raja et al., 2014), which is also positively associated with cardiovascular disease (Akil and Ahmad, 2011) and type II diabetes (Golay and Ybarra, 2005), and triglycerides, a major correlate of adipose tissue and dietary fat intake in humans (Cox and Garcia-Palmieri, 1990).

## Materials and Methods

### Participants

The study population, experimental design, and measurement protocols of the Baltimore Longitudinal Study of Aging (BLSA) have been previously reported (Ferrucci, 2008). The BLSA is a longitudinal cohort study established in 1958 and funded and conducted by the National Institute on Aging Intramural Research Program. The BLSA enrolls community-dwelling adults with no major chronic conditions or functional impairments. Usable ^1^H-MRS-based IMCL measurements (see below) were collected from 490 BLSA participants from November 2009 to September 2016. Of these, 314 participants had complete datasets including BMI, cholesterol, waist circumference, circulating triglycerides, and CT muscle attenuation measurements. Certified technicians administered all assessments using standardized protocols (Zane et al., 2017). The Institutional Review Board of the National Institute of Environmental Health Sciences approved the experimental protocol, and all participants provided written informed consent.

### Magnetic Resonance Spectroscopy

Participants were placed feet first in a 3T Philips Achieva MR scanner (Philips, Best, The Netherlands) in a supine position. IMCL values were obtained from *in vivo* spectra of ^1^H-containing metabolites using the internal body coil for excitation. A pair of Flex-M coils was used for signal acquisition, with one element placed posteriorly and one anteriorly over the vastus medialis muscle of the left mid-thigh. Localized spectra were obtained using the PRESS sequence, with a voxel size of 8 × 8 × 40 mm, with the third dimension being head-to-foot, an echo time of 55 ms, a repetition time of 2,000 ms, 32 signal averages, 4 step phase cycle, and a receiver bandwidth of 2,000 Hz. The number of complex data points in one scan, without zero-filling, was 1,024. The voxel was placed in close proximity to the femur, limiting motion and avoiding EMCL and vasculature. Voxel-specific shimming was performed using the pencil beam technique to second order. IMCL peak area was normalized to the area of the water peak (IMCL/water).

### Analysis of MRS Data

**FIGURE 1:**
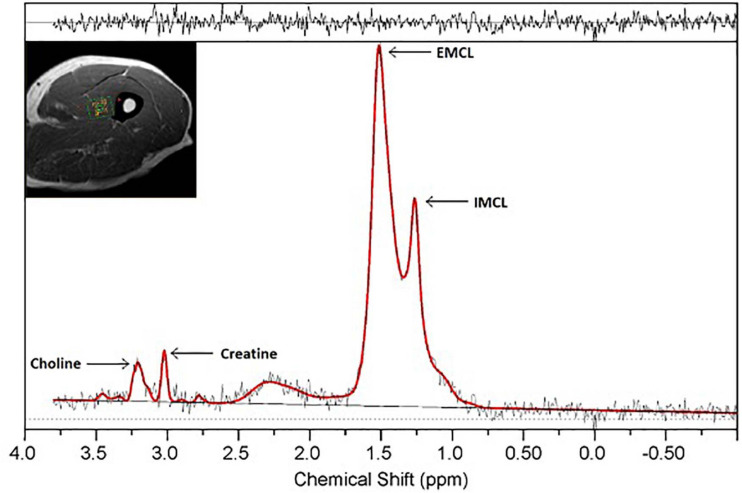
^1^H-MRS spectrum obtained from a single voxel in thigh muscle at 3.0 T. Resonances of IMCL (1.25 ppm), EMCL (1.4 ppm), choline (3.2 ppm), and creatine (3.0 ppm) are well-resolved. Clear resolution between the IMCL and EMCL resonance peaks is seen, with residuals displayed above the spectrum.

^1^H-MRS data were processed using LCModel software (Figure 1), which fits *in vivo* proton MR spectra to a linear combination of *in vitro* metabolite basis spectra (Provencher, 1993). LCModel returns residuals defining the difference between the acquired data and the modeled spectra, with larger residuals indicating inability of the model to accurately describe observed spectral amplitudes. After analysis, quality control criteria were implemented. These involved evaluating the baseline quality, residual amplitudes, and metabolite peak resolution within the acquired spectra.

A numerical value was assigned to each of these quality control measures as follows. Baseline quality was scored as 0 if a spectrum contained broad baseline components, and 1 if the baseline was sufficiently flat that it did not interfere with the measurement of the IMCL resonance amplitude. Residual amplitude was scored between 0 and 2, with 0 indicating differences >15% of peak height, 1 indicating a difference between 5 and 15% of the peak height, and 2 indicating a difference of <5%. Peak resolution was scored between 0 and 2, with 0 indicating complete lack of resolution of IMCL and EMCL peaks, 1 indicating a visible shoulder indicating the presence of two resonances, and 2 indicating peaks resolved down to 2/3 of the IMCL peak amplitude or better. Many cases that received a score of zero for peak resolution had dominant EMCL resonances, rendering quantification of the IMCL peak unreliable. Only spectra with a score of 1 or more for each criterion were eligible for analysis. This dataset consisted of 938 spectra. Of these, 299 were removed according to the exclusion criteria. Of the remaining 639 spectra, 149 were longitudinal duplicates from the same participant. After removing these, the final MRS dataset consisted of data from 490 participants. Of these, 314 subjects had CT-based muscle density measurements of the thigh, and were therefore included in the analysis, with all of these subjects also having measurements of BMI, cholesterol, waist circumference, and circulating triglycerides.

### Computerized Tomography (CT)

**FIGURE 2:**
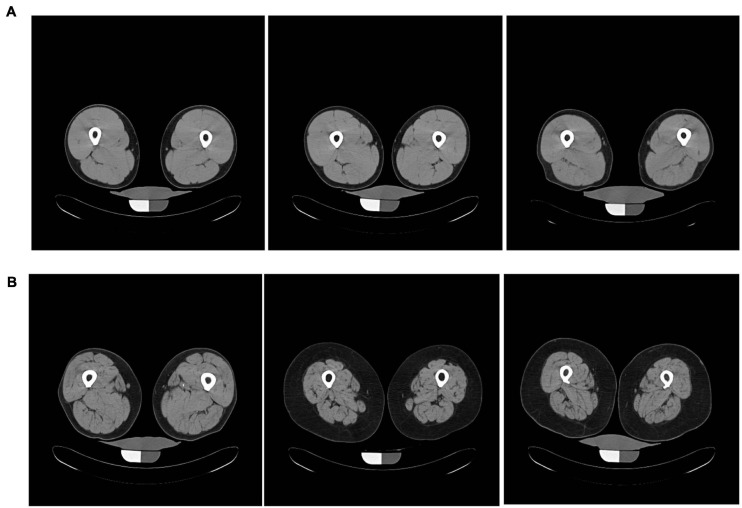
Axial mid-thigh computerized tomography images acquired from three participants with **(A)** low EMCL measurements (0.0031 ± 0.0002) and three participants with **(B)** high EMCL measurements (0.202 ± 0.085). Participants with low EMCL exhibit higher muscle CT density when compared to those with high EMCL content.

Muscle density was measured using computerized tomography (CT; Somatom Sensation 10; Siemens, Malvern, PA, United States) and quantified with BonAlyse software (Jyvaskyla, Finland). Tissue Identification and Quantification (TIDAQ) was used to generate tissue masks, which excluded intramuscular adipose tissue (IMAT). The accurately segmented compartments included air, fat, tendon, muscle, trabecular bone, and cortical bone (Makrogiannis et al., 2018). The mean density was calculated over all muscle included in the scanned slice of the mid-thigh (Figure 2). Each processed image was also visually evaluated, ensuring that there was no inaccurately identified tissue. Participants with low EMCL showed higher muscle quality and well-resolved muscle compartments when compared to participants with greater EMCL. Muscle attenuation values were reported in Hounsfield units (HU).

### Ancillary Variable Quantification

Obesity status was quantified using body mass index (BMI), which was measured as weight (kg)/height (m)^2^. Waist circumference was defined as the mean of upper abdominal circumference measurements (cm). Serum cholesterol and triglyceride concentration were measured using a standard clinical lipid panel assay. Physical activity was self-reported via a questionnaire, with values ranging from zero (sedentary) to three (very active). Physical activity scores were reported by 312 participants.

### Statistical Analysis

All statistical analyses were performed in RStudio version 1.2.1335. Data were reported as mean (standard deviation), and statistical significance was defined as *p* < 0.05.

#### Covariate Selection

In preliminary analyses, trends in the data were summarized by locally weighted scatterplot smoothing (LOWESS) (Cleveland and Devlin, 1988). In an effort to evaluate the association between IMCL/water and muscle density independent of obesity, we investigated biomarkers that would plausibly correlate with BMI. These were BMI itself, serum cholesterol, circulating triglycerides and waist circumference (Bray et al., 2018). The effects of these potential confounders were evaluated by assessing individual correlations of IMCL/water with waist circumference, circulating triglyceride concentration, BMI and serum cholesterol levels. Confounders that were found to be significantly correlated with IMCL/water were included as covariates in the final analysis; these were waist circumference, circulating triglyceride concentration and BMI.

#### Age-Adjusted IMCL

To examine the possible dependence on age of the correlation between IMCL/water and muscle density, a multivariate local polynomial regression (LOESS) analysis was performed to construct an age-adjusted IMCL variable. LOESS fits a model to a localized subset of data through multivariate smoothing and permits identification of independent variables responsible for the variation in a dependent variable.

## Results

The characteristics of the 314 participants (142 men, mean age 71.4 (12.6) years, age range 34–92 years; 172 women, mean age 70.1 (12.5) years, and age range 32–98 years) included in the analyses are reported in Table 1. Values are reflective of the good general health status of BLSA participants. The mean value of the IMCL resonance normalized by the water resonance was 0.017 (0.008), with a significant difference between men and women [women: 0.017 (0.007); men: 0.019 (0.008); *p* = 0.02]. The mean value of the EMCL resonance normalized by the water resonance was 0.038 (0.029), with a significant difference between men and women [women: 0.043 (0.025); men: 0.033 (0.031); *p* < 0.001]. The average muscle attenuation in normalized Hounsfield units was 50.5 (3.3), with a significant difference found between women and men [women: 50.0 (3.4); men: 51.0 (3.1); *p* < 0.01].

**TABLE 1:** Participant characteristics.

	*n* = 314	Men (*n* = 142)	Women (*n* = 172)	Significance (Sex)	Significance (Race)
Age (years)	70.7 ± 12.6	71.4 ± 12.6	70.1 ± 12.5	NS	N/A
BMI (kg/m^2^)	26.5 ± 4.0	27.1 ± 3.5	25.9 ± 4.3	*p* < 0.01	*p* < 0.001
Waist circumference (cm)	89.9 ± 11.4	97.1 ± 8.6	84.0 ± 10.1	*p* < 0.001	*p* < 0.05
Cholesterol (mg/dL)	180.7 ± 36.2	168.4 ± 32.8	190.9 ± 35.7	*p* < 0.001	NS
Circulating triglyceride concentration (mg/dL)	95.9 ± 47.6	103.1 ± 56.0	90.0 ± 38.3	*p* < 0.05	p < 0.001
Muscle attenuation (HU)	50.5 ± 3.3	51.0 ± 3.1	50.0 ± 3.4	*p* < 0.01	NS
IMCL/water	0.017 ± 0.008	0.019 ± 0.008	0.017 ± 0.007	*p* < 0.05	NS
Age adjusted IMCL/water	0.018 ± 0.008	0.019 ± 0.008	0.017 ± 0.007	*p* < 0.05	NS
EMCL/water	0.038 ± 0.029	0.033 ± 0.031	0.043 ± 0.025	*p* < 0.001	NS

**FIGURE 3:**
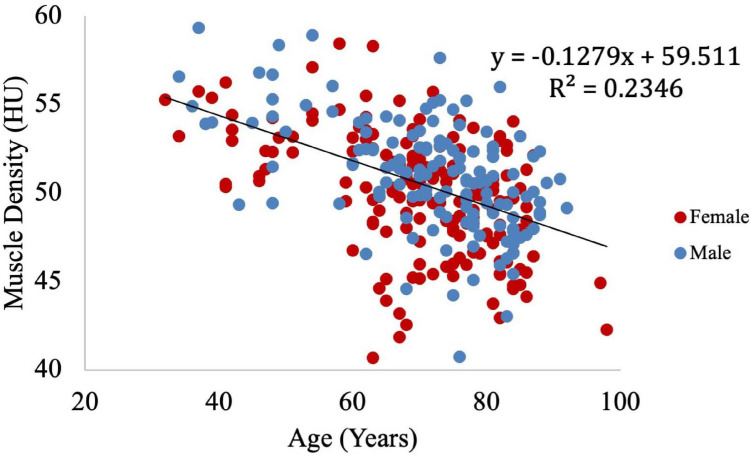
Relationship between muscle density from CT and age. As expected, muscle density decreases significantly with age (*p* < 0.001).

**FIGURE 4:**
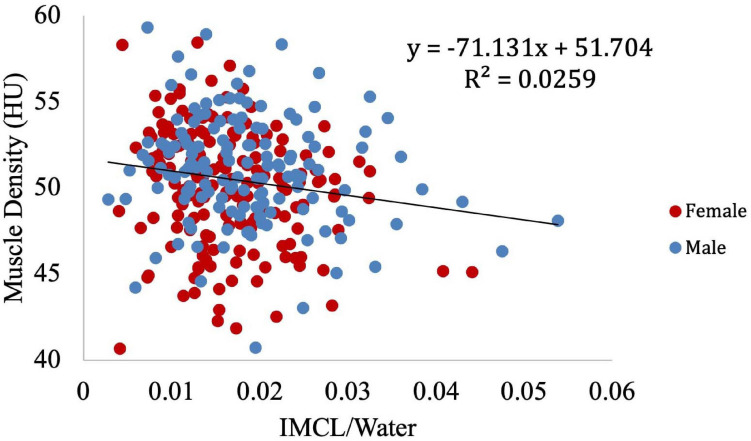
Relationship between muscle density from CT and IMCL/water (*p* < 0.001).

**FIGURE 5:**
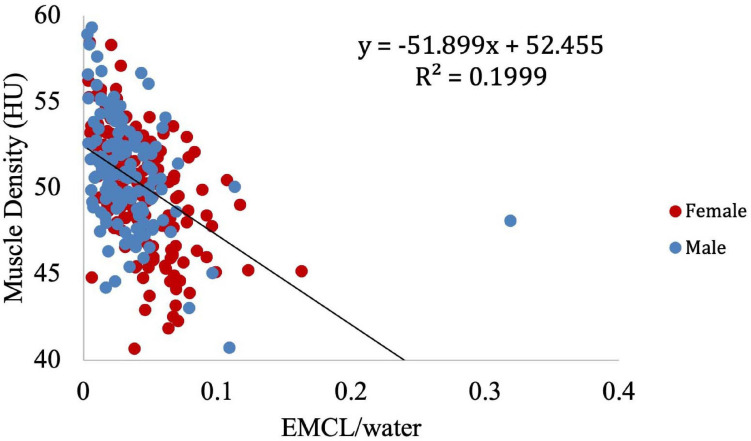
Relationship between muscle density from CT and EMCL/water (*p* < 0.001).

**FIGURE 6:**
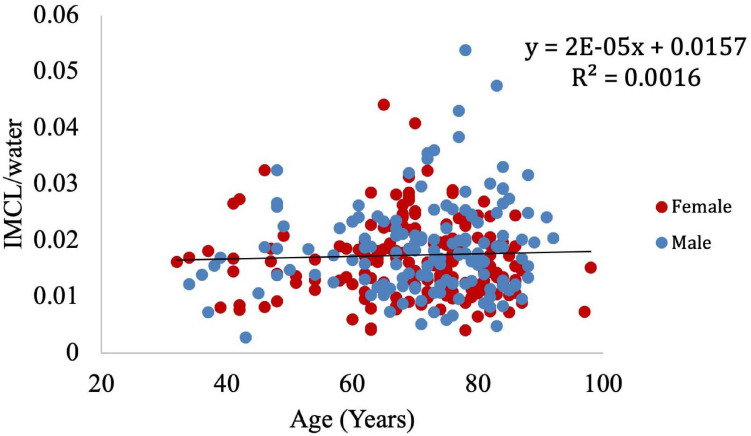
Relationship between age and IMCL/water (*p* = 0.5).

**FIGURE 7:**
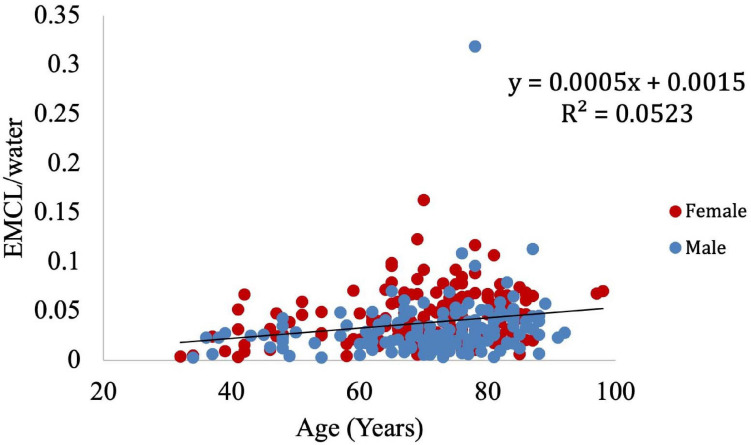
Relationship between age and EMCL/water (*p* < 0.001).

Muscle density was evaluated as a function of age (Figure 3) and exhibited a significant inverse correlation (*p* < 0.001). A significant correlation was also found between muscle density and both IMCL/water (unadjusted; Figure 4) and EMCL/water (Figure 5). Of note, IMCL/water and age were not significantly associated (Figure 6). However, EMCL/water and age were significantly associated at *p* < 0.001 (Figure 7).

**FIGURE 8:**
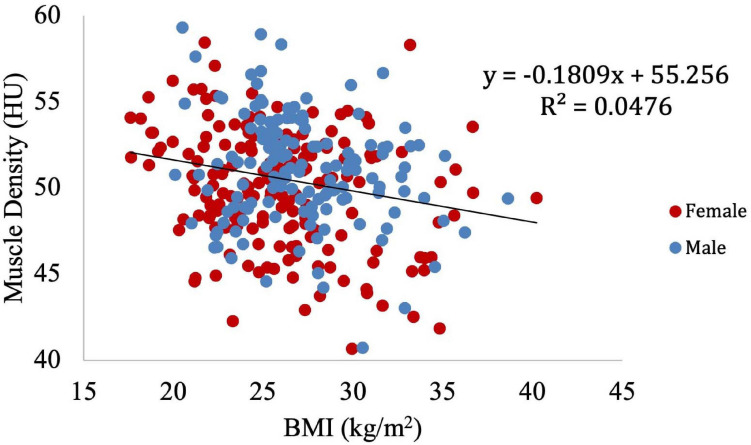
Relationship between muscle density from CT and BMI (*p* < 0.01). This relationship remained significant (*p* < 0.01) after adjustment for IMCL/water.

**FIGURE 9:**
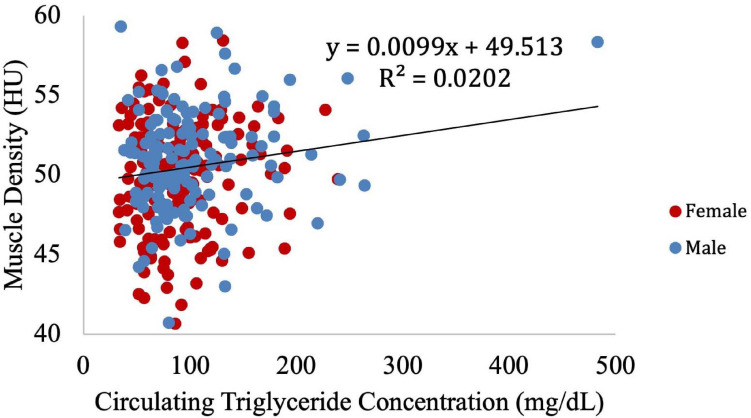
Relationship between muscle density from CT and circulating triglyceride concentration (*p* = 0.18). After adjustment for IMCL/water, the relationship between muscle density and circulating triglyceride concentration became significant (*p* = 0.04). However, when the one participant with high triglyceride concentration is removed (circulating triglyceride value of 483), this correlation is no longer significant (*p* = 0.12).

**FIGURE 10:**
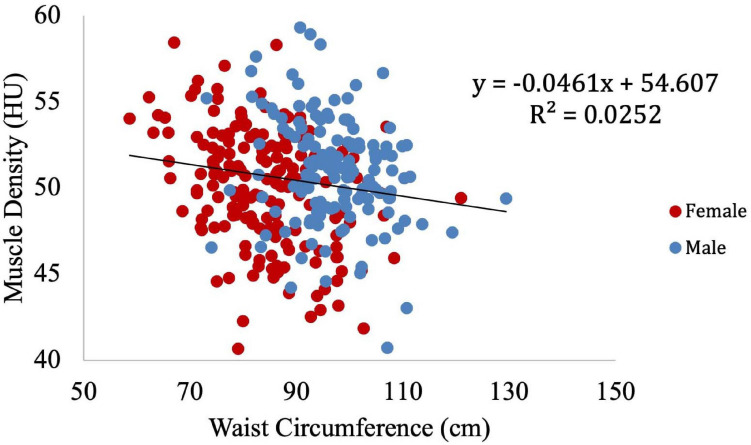
Relationship between muscle density from CT and waist circumference (*p* < 0.01). This relationship remained significant (*p* < 0.01) after adjustment for IMCL/water.

Muscle density was then independently evaluated as a function of the obesity-related variables, including BMI (Figure 8), circulating triglyceride concentration (Figure 9), and waist circumference (Figure 10). After adjusting for IMCL/water, the significant relationship between muscle density and BMI became weaker. A similar effect was found in the muscle density and waist circumference model. However, adjusting for IMCL/water increased the significance of the relationship between muscle density and circulating triglyceride concentration.

In order to identify potential confounders of the muscle density and IMCL relationship, we examined clinical obesity-related variables. Cholesterol was not significantly associated with IMCL/water and was excluded as a covariate, while BMI, waist circumference, and circulating triglyceride concentration were significantly correlated with IMCL/water and therefore included as covariates (Table 2).

**TABLE 2:** Multivariate analysis adjusting for confounding variables.

	Model 1: MD = IMCL		Model 2: MD = IMCL + Race + Sex		Model 3: MD = IMCL + Race + Sex + Obesity	
Parameters	β (95% CI)	*P*-value	β (95% CI)	*P*-value	β (95% CI)	*P*-value
IMCL/water	−62.64 (−84.28, −41.00)	0.004**	−76.48(−97.70, −55.26)	< 0.001***	−60.61 (−81.75, −39.47)	0.004**
Age	−0.126 (−0.139, −0.113)	< 0.001***	−0.124 (−0.136, −0.111)	< 0.001***	−0.120 (−0.132, −0.108)	< 0.001***
Race	−	−	0.248 (0.097, 0.399)	0.101	−0.019 (−0.171, 0.133)	0.899
Sex	−	−	1.19 (0.861, 1.52)	< 0.001***	1.92 (1.48, 2.36)	< 0.001***
Physical activity	−	−	0.390 (0.209, 0.571)	< 0.05*	0.278 (0.103, 0.453)	0.113
BMI (kg/m^2^)	−	−	−	−	−0.082 (−0.152, −0.012)	0.238
Waist Circumference (cm)	−	−	−	−	−0.061 (−0.091, −0.031)	0.041*
Circulating triglyceride concentration (mg/dL)	−	−	−	−	0.012 (0.009, 0.015)	< 0.001**

IMCL/water is a significant covariate in the relationship between muscle density (MD) and age, indicating that increased IMCL may play a significant role in this relationship. ***p < 0.001,**p < 0.01, and *p < 0.05. Physical activity data were available for 312 participants.

After introducing age as a potential confounder in the regression model (Table 2: Model 1), the significant relationship between muscle density and IMCL/water was retained, and the inverse relationship between age and muscle density remained statistically significant. After further adjustment for physical activity, sex and race (Table 2: Model 2), the independent relationship between IMCL/water and muscle density remained statistically significant.

The role of obesity in the relationship between IMCL and muscle density was also investigated (Table 2: Model 3); with waist circumference being significantly associated with a lower muscle density. Surprisingly, circulating triglyceride concentration exhibited a significant positive correlation with muscle density. The analysis showed no significant interaction between CT values for muscle attenuation and BMI. After adjusting for age, sex, race, physical activity, BMI, circulating triglyceride concentration, and waist circumference, the relationship between IMCL/water and muscle density remained significant (*p* < 0.01).

**FIGURE 11:**
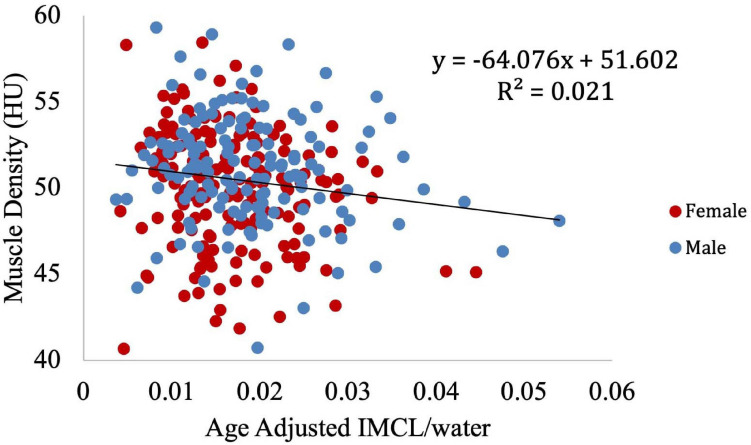
Correlation between age-adjusted IMCL/water ratio obtained from MR spectroscopy and muscle density obtained from CT. As shown, decreased density was associated with increased age adjusted IMCL/water (*p* < 0.01). The age adjusted IMCL/water values were calculated using a LOESS regression.

To further visualize the relationship between IMCL and muscle density, LOESS regression was used to construct an age-adjusted IMCL/water variable. Muscle density was then evaluated as a function of age-adjusted IMCL/water (Figure 11). As in the non-adjusted analysis, greater age-adjusted IMCL/water was significantly associated with lower muscle density (*p* < 0.01; Table 3: Model 1). Additionally, circulating triglyceride concentration was found to be significantly and positively correlated with muscle attenuation, while waist circumference showed a significant negative correlation. No significant correlation was found between muscle attenuation and BMI. After adjusting for all covariates mentioned above, the relationship between age-adjusted IMCL/water and muscle density remained significant (*p* < 0.05), suggesting that the association is independent of physical activity level, sex, race (Table 3: Model 2), and general obesity markers (Table 3: Model 3). The association was linear across the muscle attenuation range of 40.7–59.3 normalized Hounsfield units.

**TABLE 3:** Multivariate analysis adjusting for confounding variables.

	Model 1: MD = Age Adjusted IMCL		Model 2: MD = Age Adjusted		Model 3: MD = Age Adjusted IMCL +	
			IMCL + Race + Sex		Race + Sex + Obesity	
Parameters	β (95% CI)	*P*-value	β (95% CI)	*P*-value	β (95% CI)	*P*-value
Age adjusted IMCL/water	−64.08 (−88.84, −39.32)	0.010*	−75.79 (−100.13, −51.45)	< 0.01**	−60.11 (−84.36, −35.86)	0.014*
Race	−	−	0.263 (0.090, 0.436)	0.123	−0.041 (−0.216, 0.134)	0.816
Sex	−	−	0.914 (0.539, 1.29)	< 0.05*	2.13 (1.63, 2.63)	< 0.001***
Physical activity	−	−	0.664 (0.459, 0.869)	< 0.01**	0.551 (0.353, 0.749)	< 0.01**
BMI	−	−	−	−	0.032 (−0.047, 0.111)	0.690
Waist circumference (cm)	−	−	−	−	−0.111 (−0.145, −0.077)	< 0.001***
Circulating triglyceride concentration (mg/dL)	−	−	−	−	0.015 (0.011, 0.019)	< 0.001***

Age Adjusted IMCL/water is significantly associated with lower muscle density, independent of additional obesity markers. ***p < 0.001, **p < 0.01, and *p < 0.05. Physical activity data were available for 312 participants.

## Discussion

We found a statistically significant inverse relationship between IMCL/water ratio and muscle density. This relationship was maintained after formal adjustment for demographic and obesity-related variables. Our findings are consistent with those reported by Goodpaster et al. (2000a) in which muscle attenuation values from CT were negatively correlated with skeletal muscle lipid content, as determined by muscle biopsy followed by quantification of intramuscular fat with histochemical analysis. While causation was not addressed in our work, the reported correlations suggest a hypothesis that the accumulation of IMCL may be a significant contributor to the reduction of muscle density with aging. This would form the basis for further investigation through a longitudinal study.

Aging is associated with decreased muscle strength, mass and density (Doherty, 2003; Newman et al., 2005; Kuk et al., 2009; Miljkovic and Zmuda, 2010). The decline in strength contributes to the diminished physical performance with aging (Brill et al., 2000). Given the established correlation between muscle density and strength, IMCL accumulation may contribute directly to the decrease in muscle strength and function with age (Siparsky et al., 2014), in addition to its likely causative role in metabolic disorders.

Our results are important because while muscle density is a crucial correlate of the disabling effect of aging and many chronic diseases and is associated with increased hospitalizations in older persons, the mechanistic underpinnings of decreased muscle density remain elusive (Cawthon et al., 2009; Delmonico et al., 2009; Demontis et al., 2013). Aside from its role in aging, low muscle density has been shown to correlate with other pathology, including rheumatoid arthritis (RA), a systemic inflammatory condition that can result in disability and joint deformity. In particular, CT-determined thigh muscle density was found to correlate with increased serum interleukin-6 levels, glucocorticoid requirement, number of joints exhibiting tenderness, and disease progression (Kramer et al., 2012). Muscle density is also associated with physical function in idiopathic inflammatory myopathy (IIM) patients. In these individuals, low muscle density is correlated with a decrease in physical function, endurance, and strength (Cleary et al., 2015). In these studies, however, the potential role of fat infiltration as a causative factor in lower muscle density was not investigated. Low muscle density is also considered a biomarker of impaired metabolism and it is often observed in people with increased insulin resistance and metabolic syndrome (Kim et al., 2003). Similarly, increased levels of IMCL are associated with insulin resistance in obese patients (Pan et al., 1997). Insulin resistance in muscle is in part characterized by decreased oxidative capacity and impaired fatty acid oxidation (Goodpaster et al., 2001b). Our findings reinforce the hypothesis that IMCL accumulation in skeletal myocytes is an essential part of the reduction of muscle density with aging and not merely the result of the confounding effects of body composition changes. Whether interventions that diminish IMCL infiltration also affect muscle quality, improve metabolic control and reduce insulin resistance remains an important topic for future studies.

We note that EMCL is expected to dominate IMCL in terms of overall fat content, even when macroscopic fat deposits are excluded. Accordingly, CT-based muscle density would depend more strongly on EMCL. However, as outlined above, EMCL, being much less readily accessible to intracellular machinery, is not as metabolically active as IMCL. Thus, in spite of its weaker correlation with HU, it is IMCL in which we are primarily interested. We have found this correlation to be operative on a population-based scale, though not on the scale of an individual subject.

Although, as far as we know, this is the largest study to date using ^1^H-MRS to quantify IMCL in muscle, certain limitations remain. Accurate quantification of IMCL by ^1^H-MRS requires strict attention to spectral quality. We ensured the accurate measurement of IMCL using ^1^H-MRS through application of specific quality control criteria. The most common reason for excluding a spectrum from our analysis was an absence of clear resolution between the IMCL and EMCL peaks, in which case IMCL cannot be accurately quantified. This lack of resolution was most often due to a large EMCL resonance from the selected spectral voxel, indicating high EMCL content. However, rejection of these spectra may have also excluded those with high IMCL concentrations, limiting our range in IMCL/water ratio. Nevertheless, this range is comparable to those found in the literature. Additionally, the presence of macroscopic, “marbled” fat can result in poorly resolved separation between IMCL and EMCL peaks. Removing these spectra from analysis has the potential to introduce bias through exclusion of individuals with a greater degree of obesity. Indeed, BMI data were available for 297 participants for which spectra were excluded. The average BMI of these participants was 27.4 ± 3.9, compared to an average BMI of 26.5 ± 4.0 for included participants. While this difference in BMI did reach statistical significance (*p* < 0.01), the difference in absolute terms was small (∼3%).

Since ^1^H-MRS only provides normalized metabolite concentrations, we were unable to determine absolute water concentrations. However, previous work has indicated that the ratio IMCL/water was significantly correlated with IMCL normalized to an external oil phantom (Larson-Meyer et al., 2006). This supports the use of quantitative IMCL/water measurements in our analysis.

Voxel placement considerations represent a further limitation of our study. While the majority of participants had IMCL data obtained from the vastus medialis region of the lower extremity, data were collected from regions closer to the vastus intermedius for participants with large amounts of EMCL, as the vastus medialis was too small to contain the voxel without encroaching on macroscopic fat deposits. In these individuals with greater macroscopic fat, there was particularly poor resolution between these two muscles, making it very difficult to specify which provided the predominant contribution to the MRS signal. In addition, the CT imaging voxels and the corresponding MRI spectroscopic voxels could be co-localized, but not precisely registered. Briefly, the MRS technique used is a localizing technique, but not an imaging technique. The much larger MRS voxels will exhibit partial overlap with several CT pixels, and will not be registered with them, so there will not be a simple correspondence between the contents of the MRS voxel and a set of CT pixels. Moreover, the CT slice of 10 mm was considerably thinner than the MRS voxel, which was up to 40 mm long in the inferior-superior direction, depending on voxel orientation. Thus, the MRS values will include contributions from regions not covered by CT. Nevertheless, while we cannot register on a pixel-by-pixel basis, we did obtain data from the same region using the two techniques to achieve the desired correspondence.

Use of the BLSA population enabled us to perform measurements in a relatively well-controlled healthy cohort across a large age range but may limit the applicability of these results to more typical populations. Nevertheless, our methods permitted an assessment of the correlation between IMCL and the decrease in muscle density with age, which is expected to be generalizable. The present correlative findings support further investigation of the hypothesis that increased concentration of IMCL is associated with poor muscle quality; our results are consistent with but greatly extend previous findings (Larson-Meyer et al., 2006).

In sum, we find that the correlation between IMCL and functional biomarkers, and in particular muscle density assessed through CT, cannot be meaningfully established in an individual subject. However, population-based IMCL measurement may serve as an indicator of metabolic and functional health in the aging population.

## Data Availability Statement

The raw data supporting the conclusions of this article will be made available by the authors, without undue reservation.

## Ethics Statement

The studies involving human participants were reviewed and approved by the Ethics Branch, National Institute on Aging/NIH, Bethesda, MD. The patients/participants provided their written informed consent to participate in this study.

## Author Contributions

NB, KF, DR, RS, and LF conceived the study. NB, RS, and LF analyzed the data gathered, performed statistical analysis, and drafted the manuscript. All the authors contributed to the manuscript editing, and read and approved the submitted version.

## Conflict of Interest

The authors declare that the research was conducted in the absence of any commercial or financial relationships that could be construed as a potential conflict of interest.
